# Live attenuated *Salmonella* typhimurium vaccines delivering SaEsxA and SaEsxB via type III secretion system confer protection against *Staphylococcus aureus* infection

**DOI:** 10.1186/s12879-018-3104-y

**Published:** 2018-04-25

**Authors:** Chen Xu, Bao-zhong Zhang, Qiubin Lin, Jian Deng, Bin Yu, Smriti Arya, Kwok-Yung Yuen, Jian-Dong Huang

**Affiliations:** 1 School of Biomedical Sciences, The University of Hong Kong, Hong Kong, SAR China; 2 Department of Microbiology, The University of Hong Kong, Hong Kong, SAR China; 3 HKU-Shenzhen Institute of Research and Innovation, Shenzhen, China; 4 Shenzhen Institute of Advanced Technologies, Shenzhen, China

**Keywords:** Live attenuated *S.* Typhimurium vaccine, T3SS, MRSA, *S. aureus*, SaEsxA, SaEsxB

## Abstract

**Background:**

*Staphylococcus aureus (S. aureus)* causes a wide range of infectious diseases in human and animals. The emergence of antibiotic-resistant strains demands novel strategies for prophylactic vaccine development. In this study, live attenuated *S. enterica* subsp. *enterica* serotype Typhimurium *(S.* Typhimurium*)* vaccine against *S. aureus* infection was developed, in which *Salmonella* Pathogenesis Island-1 Type 3 Secretion System (SPI-1 T3SS) was employed to deliver SaEsxA and SaEsxB, two of ESAT-6-like (Early Secreted Antigenic Target-6) virulence factors of *S. aureus*.

**Methods:**

Antigens SaEsxA and SaEsxB were fused with the N-terminal secretion and translocation domain of SPI-1 effector SipA. And cytosolic delivery of Staphylococcal antigens into macrophages was examined by western blot. BALB/c mice were orally immunized with *S.* Typhimurium-SaEsxA and *S.* Typhimurium-SaEsxB vaccines. Antigen-specific humoral and Th1/Th17 immune responses were examined by ELISA and ELISPOT assays 7–9 days after the 2nd booster. For ELISPOT assays, the statistical significance was determined by Student’s t test. The vaccine efficacy was evaluated by lethal challenge with two *S. aureus* clinical isolates Newman strain and USA 300 strain. Statistical significance was determined by Log rank (Mantel-Cox) analysis. And a *P* value of < 0.05 was considered statistically significant.

**Results:**

Oral administration of *S.* Typhimurium-SaEsxA and *S.* Typhimurium-SaEsxB vaccines induced antigen-specific humoral and Th1/Th17 immune responses, which increased the survival rate for vaccinated mice when challenged with *S. aureus* strains.

**Conclusions:**

The newly developed *S.* Typhimurium-based vaccines delivering SaEsxA and SaEsxB by SPI-1 T3SS could confer protection against *S. aureus* infection. This study provides evidence that translocation of foreign antigens via *Salmonella* SPI-1 T3SS into the cytosol of antigen presenting cells (APCs) could induce potent immune responses against pathogens.

**Electronic supplementary material:**

The online version of this article (10.1186/s12879-018-3104-y) contains supplementary material, which is available to authorized users.

## Background

*Staphylococcus aureus*, a commensal of human skin and nares, is also a human pathogen with dual intracellular and extracellular lifestyle [1, 2]. *S. aureus* infection causes a broad range of diseases, from skin infections to life-threatening diseases, including pneumonia, endocarditis, and sepsis et al. [1, 3]. The recent emergence of multidrug-resistant *S. aureus* strains, such as methicillin-resistant *S. aureus* (MRSA), exerts a huge clinical burden and makes the infections much more difficult to treat [4–6]. However, a prophylactic vaccine against *S. aureus* is not available. Many active and passive immunization vaccines, such as StaphVAX, V710, Pagibaximab, tefibazumab, Veronate, Aurograb and AltaStaph, ended in failure in clinical trials [7–13], which might be attributed to several reasons. Firstly, cell-surface antigens were targeted by all the seven vaccines, and the elicited antibodies against surface components might promote bacterial aggregation, causing possible tissue damage, ischaemia, multi-organ failure and death [7, 14]. Secondly, failures of the five passive immunization strategies might be ascribed to an overemphasis on humoral immunity, rather than the cooperation of humoral and cellular immunity [7, 15].

Secreted virulent factors are also targeted for vaccine development and exhibit effective protection against *S. aureus* infection diseases [16–18]. Among them, SaEsxA and SaEsxB are two ESAT-6-like virulence factors required for persistence and spread of *S. aureus* in the infected host [19]. SaEsxA and SaEsxB were first identified in the culture filtrates of *Mycobacteria tuberculosis (M. tuberculosis)* and found to be able to stimulate T-cell immune responses [20, 21]. In 2005, SaEsxA and SaEsxB were experimentally verified to be secreted from *S. aureus* and involved in abscess formation. *S. aureus* mutant lacking EsxA or EsxB showed defects during *S. aureus* infection in murine model [19]. SaEsxA and SaEsxB also regulate the apoptosis and release of intracellular *S. aureus* from the infected epithelial cells [2].

During recent years, protein subunit vaccines targeting SaEsxA and SaEsxB were shown to prevent invasive *S. aureus* infection through induction of Th1- and Th17-biased immunity [22]. Administration of a four-component vaccine 4C-Staph/alum including SaEsxA/B chimera was demonstrated to protect mice from *S. aureus* infection in four murine models, including kidney abscess, peritonitis, skin and pneumonia models [23, 24]. In addition, a novel adjuvant T7-alum targeting TLR7 was formulated with the four component vaccine 4C-Staph (4C-Staph/T7-alum), which showed outperformance over the previous 4C-Staph/alum [25]. The immunologic protection was attributed to the cooperation of vaccine-specific antibodies, CD4+ T cells and IL-17 [25]. This study emphasizes on the cooperation of humoral and cellular immunity for protection against *S. aureus* infection.

Live attenuated *S.* Typhimurium strains have been employed to deliver recombinant foreign antigens with various approaches [26]. Among these, SPI-1 T3SS has been engineered for the cytosolic delivery of foreign antigens to induce efficient immune responses against cancer and infectious diseases [27, 28]. SPI-1 T3SS-mediated cytosolic delivery of antigens could significantly enhance the accessibility of antigens to MHC class-I antigen presenting pathway, and effectively activate CD8+ T cell-mediated immunity, which is important to remove intracellular pathogens such as *S. aureus* [27, 29–31]. In addition, utilization of *Salmonella* T3SS for protein expression can prevent formation of inclusive bodies and cellular degradation of target proteins, which happened during protein expression by other gram-negative expression systems [32].

SPI-1 T3SS is a needle-like apparatus to directly translocate effectors into mammalian host cells, which facilitates bacteria invasion and pathogenesis [33–35]. This process is mediated by different chaperones targeting the N-terminal tags of the cognate effectors [36]. In this study, N-terminal domain of SipA (1-169aa) was fused with staphylococcal antigens to achieve the translocation. SipA is a bi-functional SPI-1 T3SS effector. On one hand, SipA functions extracellularly to mediate trans-epithelial migration of polymorphonuclear neutrophils [37], via engaging certain host surface receptors to activate protein kinase C (PKC) and subsequently induce the apical release of pathogen-elicited epithelial chemoattractant (PEEC) [37–39]. On the other hand, SipA could also be translocated into the cytosol of host cells by T3SS, inducing actin cytoskeleton rearrangement and membrane ruffle, which are required for bacterial uptake [40, 41].

In this study, SPI-1 T3SS was utilized to deliver Staphylococcal antigens SaEsxA and SaEsxB into the cytosol of host cells. Our results showed that oral immunization with these strains could elicit multifaceted immune responses in mice, which conferred protection against *S. aureus*. To the best of our knowledge, this is the first development of live attenuated *S.* Typhimurium based vaccine against *S. aureus* infection. Besides, this study also provides information that N-terminal signal peptide of SipA (1-169aa) could be utilized as molecular carrier for the cytosolic delivery of foreign proteins via SPI-1 T3SS, with the support of the cognate chaperon invB.

## Methods

### Bacterial strains and growth conditions

Bacteria strains and plasmids used in this study were listed in Table 1.

**Table 1:** Bacterial strains and plasmids used in this study

Strains	Description	Reference
SL7207	ΔaroA	Lab stock
ML21	ΔaroA ΔpyrF	Lab stock
ML86	ML21 ΔsipB	Lab stock
ML88	ML21 ΔinvA	Lab stock
N19	ML21 carrying PagC-invB-sipA-SaEsxA (ColE1 ori)	This study
N20	ML21 carrying PagC-invB-sipA-SaEsxB (ColE1 ori)	This study
N106	ML21 carrying empty plamsid (ColE1 ori)	This study
N80	ML86 (ΔsipB) carrying PagC-invB-sipA-SaEsxA (ColE1 ori)	This study
N158	ML88 (ΔinvA) carrying PagC-invB-sipA-SaEsxA (ColE1 ori)	This study
N160	ML86 (ΔsipB) carrying PagC-invB-sipA-SaEsxB (ColE1 ori)	This study
N161	ML88 (ΔinvA) carrying PagC-invB-sipA-SaEsxB (ColE1 ori)	This study

*Salmonella enterica serovar* Typhimurium aroA-deleted (ΔaroA) strain SL7207 was kindly provided by Dr. B.A.D Stocker [42], which was utilised as the parent for genomic knockouts. ML21 is an isogenic double knockout (ΔaroA, ΔpryF), derived from SL7207. ML88 is a triple knockout (ΔaroA, ΔpryF, ΔinvA), derived from ML21. The isogenic knockouts were constructed using λ Red-recombineering method [43].

*S.* Typhimurium and *E. coli* strains were cultured in Luria-Bertani (LB) broth or LB agar supplemented with appropriate antibiotics. For secretion assay, *S.* Typhimurium was cultured in SPI-1 Inducing LB (0.3 M NaCl) [36]. *S. aureus* strains were grown in Brain Heart Infusion Broth (BHI, Sigma-Aldrich) and BHI agar.

Antibiotics were used at the final concentrations: streptomycin (50 μg/mL), chloramphenicol (25 μg/mL), ampicillin (100 μg/mL) and kanamycin (50 μg/mL).

### Mammalian cell culture

Murine RAW264.7 macrophages were purchased from ATCC and cultured in RPMI 1640 medium (Gibco) with 10% Fetal Bovine Serum (FBS, Gibco). Cell cultures were maintained at 37 °C in a humidified atmosphere containing 5% CO_2_.

### Construction of plasmids

Plasmid pCASP (-sicP-sptP) (Genbank: #EF179157), which is based on pPROTet.133 backbone (Cm^R^, ColE1) (BD Clonetech), was generously provided by Prof. Christopher Voigt [36]. Plasmids with alternative secretion tag/chaperone pairs, including pCASP-invB-sipA, pCASP-invB-sopE2, pCASP-invB-sopA, and pCASP-sigE-sopB were constructed by overlap extension PCR, as previously described [36]. Briefly, secretion tags, chaperones and PsicA were amplified from *S.* Typhimurium genomic DNA with the primers listed in Table 2. The pcr products were fused by overlap extension PCR, followed by double enzyme digestion (HindIII and XhoI) and ligation into the plasmid pCASP to replace PsicA-sicP-sptP. For the simplicity of further genetic modification, PacI restriction enzyme site was introduced between PsicA and chaperones.

**Table 2:** Primers used in this study

Primer Name	Primer Sequences (5′ to 3′)	Purposes
BamHI-SaEsxA-F	CGGGATCCATGGCAATGATTAAGATG	amplify SaEsxA-his
NotI-SaEsxA-his-taa-R	ATAAGAATGCGGCCGCTTAATGATGATGATGATGATGTTGCAAACCGAAATTATTAGAAAGTTGTTG	amplify SaEsxA-his
HindIII-SaEsXB-F	CCCAAGCTTGGTGGATATAAAGGTATTAAAGCAGATG	amplify SaEsxB-his
NotI-SaEsxB-his-taa-R	ATAAGAATGCGGCCGCTTAATGATGATGATGATGATGTGGGTTCACCCTATCAAGCC	amplify SaEsxB-his
XhoI-PagC-F	CCGCTCGAGGTTAACCACTCTTAATAATAATGGGTTTTATAGC	amplify PpagC
PacI-PagC-R	CCTTAATTAATACTACTTATTATTTACGGTGTGTTTAAACAC	amplify PpagC
check-pCASP-R	CCGCCTTTGAGTGAGCTGATAC	check sequence from the down stream of heterologous genes
check-PsicA-F	CGATCAACGTCTCATTTTCGCC	check sequence from the upstream of PsicA
XhoI-PsicA-F	CTCGAGCCACAAGAAAACGAGG	amplify PsicA
PsicA-R (no sicA rbs)	CACCGACTTTGTAGAACTTAACG	amplify PsicA
(PsicA)invB-F	CGGTGACAGATAACAGGAGTAAGTATTAATTAAGGAAAAGATCTATGCAACATTTGG	amplify (PsicA)-invB
invB-R (sipA)	CACTTGTAACCATTATTAATATCCTCTTCTGTTATCTCATTAGCGACCGACTAAAAAC	amplify (PsicA)-invB-(sipA)
invB-R(sopE2)	CATTTTCTCCTCTTTAATTTATCTCATTAGCGACCGACTAAAAAC	amplify (PsicA)-invB-(sopE2)
invB-R	ATCTCATTAGCGACCGACTAAAAAC	amplify (PsicA)-invB
(invB)-sipA-F	GTTTTTAGTCGGTCGCTAATGAGATAACAGAAGAGGATATTAATAATGGTTACAAGTG	amplify (invB)-sipA
HindIII-sipA-R	CCCAAGCTTTCCTGACTGAAAATACAAATTCTCTCCACCGCCAGTGTTATTTTTGATAATATCTAAC	amplify (invB)-sipA
(invB)-sopE2-F	GATAAATTAAAGAGGAGAAAATGACTAACATAACACTATCCACCCAG	amplify (invB)-sopE2
HindIII-sopE2-R	AAGCTTTCCTGACTGAAAATACAAATTCTCTCCGGCCGGATCTTTACTCGC	amplify (invB)-sopE2
(invB)-sopA-F	GTTTTTAGTCGGTCGCTAATGAGATaaTTGATAAGGAATTGTAATGAAGATATCATCAGG	amplify (invB)-sopA
HindIII-sopA-R	AAGCTTTCCTGACTGAAAATACAAATTCTCTCCCTTGCCTGCATTATTTGTATCTTTAATATTTTTAAC	amplify (invB)-sopA
(sicA)-sipC-F	GTGAACAAGAAAAGGAATAATAAAGGGAGAAAAATATGTTAATTAGT	amplify (sicA)-sipC
HindIII-sipC-R	AAGCTTTCCTGACTGAAAATACAAATTCTCTCCTCCGCTAATATCAAAAAACTTTCCGAC	amplify (sicA)-sipC
(PsicA)sigE-F	CGTTAAGTTCTACAAAGTCGGTG GAGTCTTGAGGTAACTATATGGAAAGTC	amplify (PsicA)-sigE-(sopB)
sigE-R-(sopB)	CCTGATTATGCATAATGCTCTTTCAATTGCTTC	amplify (PsicA)-sigE-(sopB)
(sigE)sopB-F	GAGCATTATGCATAATCAGGAATATTAAAAACGCTATGCAAATAC	amplify (sigE)-sopB
HindIII-sopB-R	AAGCTTTCCTGACTGAAAATACAAATTCTCTCCGTTATTAAGCTGCTTGACCTGAGC	amplify (sigE)-sopB
pyrF-5’	ATCCAATTTGCGCCACTTCCGGTGCCCATCATCAAGAAGGTCTGGTCATGCCGATCATATTCAATAACCCT	knockout pyrF gene
pyrF-3’	CCCCGTCTGCGTTGAATAAACCAGACGACTATTGGAATCGCTCATTATGCGACTAGTGAACCTCTTCGAGGG	knockout pyrF gene
check-pyrF-R	CGGTATCGTTGTCAGAAATGCGGT	check pyrF deletion
check-pyrF-R	CGTGATTGGTCACCAGGTTGGAAA	check pyrF deletion
invA-5’	GCAGAACAGCGTCGTACTATTGAAAAGCTGTCTTAATTTAATATTAACAGGATACCTATAGCCGATCATATTCAATAACCCT	knockout invA gene
invA-3’	CGGAACGAACTAATTCAGCGATATCCAAATGTTGCATAGATCTTTTCCTTAATTAAGCCCGACTAGTGAACCTCTTCGAGGG	knockout invA gene
check-invA-F	TTACCAAAGCGTTTAATGCG	check invA deletion
check-invA-R	CATCCTTCCATTATGGTCAT	check invA deletion

SaEsxA and SaEsxB were PCR amplified from the genomic DNA of *S.aureus* ATCC 25923 with primers including 6 × his tag. After double enzyme digestion (BamHI and NotI), DNA fragments SaEsxA-his and SaEsxB-his were ligated into the BamHI and NotI restriction sites of pCASPs in frame with different signal peptides.

The pagC promoter (PpagC) was PCR amplified from the genomic of *S.* Typhimurium genomic DNA. After digested with restriction enzyme XhoI and PagC, PpagC was inserted into plasmids PsicA-invB-sipA-SaEsxA and PsicA-invB-sipA-SaEsxB to replace PsicA.

### Secretion assays

Secretion assay was performed according to the method described previously with modifications [36]. Briefly, bacteria strains were streaked on LB agar plates supplemented with appropriate antibiotics and cultured at 37 °C overnight. Single colonies were inoculated in 4 mL Luria-Bertani (LB) broth media, supplemented with chloramphenicol and streptomycin and grown overnight at 220 rpm. The next morning, bacteria cultures were diluted to an OD600 of 0.01 in 4 mL fresh LB broth, supplemented with chloramphenicol and streptomycin and grown for 2 h at 220 rpm. Then the bacteria cultures were diluted at the ratio of 1:10, into 4 mL inducing LB (0.3 M NaCl) supplemented with chloramphenicol and streptomycin, and were grown for 8 h at 160 rpm. Media and bacteria were separated by centrifugation at 4700 g for 10 min. Supernatants were filtered with 0.22 μm syringe filters (Sartorius). The supernatant samples were concentrated 20X by trichloroacetic acid (TCA) precipitation. Briefly, 100% (*w*/*v*) TCA was added to the supernatants to a final concentration of 20%, and incubated at 4 °C overnight. The mixture was centrifuged at 17,000 g for 5 min at 4 °C. And the pellet was washed with 500 μL cold acetone. Pellets were dried at room temperature for about 5 min and loaded onto sodium dodecyl sulfate polyacrylamide gel electrophoresis (SDS-PAGE) after re-suspended in 1× SDS-PAGE loading buffer. On the other hand, the bacteria pellets were washed with cold PBS once and re-suspended in PBS containing proteinase inhibitors, which is a mixture of Aprotinin from bovine lung (Sigma), Leupeptin hydrochloride (Sigma) and Pepstatin A (Sigma). The bacterial cells were sonicated for 2 min in 4 s pulses with 35% amplitude, followed by centrifugation at 19,000 g for 20 min. The supernatants were collected, and mixed with 6× SDS-PAGE loading buffer for SDS-PAGE.

### Bacterial infection of RAW264.7 macrophages and translocation assays

The detection of translocated heterologous antigens was carried out as described previously with modifications [44]. Briefly, bacteria were cultured overnight at 37 °C in LB broth supplemented with appropriate antibiotics, and subcultured at 1:30 dilution for 2 h until OD600 reached 1.5. Then bacteria were harvested by centrifugation at 17,000 g for 2 min, washed once with sterile PBS, and re-suspended in RPMI 1640 medium with 10% FBS. When Raw264.7 macrophages reached to 70–80% confluency in 60 mm tissue culture dishes, bacteria were added at a MOI of 100 and infected the cells at 37 °C for 3 h in 3 mL RPMI 1640 medium with 10% FBS. After infection, cells were washed thrice with PBS, and cultured in RPMI 1640 medium with 10% FBS containing 100 μg/mL gentamicin for another hour. After washed with cold PBS once, cells were lysed by lysis buffer which is PBS containing 0.1% Triton X-100 and proteinase inhibitors, and detached with a rubber cell scraper. Cell soluble fraction (containing bacteria-free cytosol) and insoluble fraction (containing insoluble cell components and intracellular bacteria) were separated by centrifugation at 19,000 g for 20 min. The soluble fraction samples were further filtered by 0.22 μm filter (Sartorius) to get rid of bacteria, and then concentrated by TCA precipitation, as described above. Both soluble and insoluble fractions were used for western blot analysis.

### SDS-PAGE and western blot

SDS-PAGE and western blot was conducted following standard methods. Protein samples were mixed with SDS-loading dye, boiled for 10 min and analyzed by 12% SDS-PAGE. Separated proteins were transferred to PVDF membrane and blocked by 5% *w*/*v* skim milk/TBST (Tris Buffered Saline containing 0.1% Tween 20) for 1 h at room temperature. Membranes were incubated with mouse anti-6 × His tag monoclonal antibody (HIS.H8, MA1–21315) at 1:2000 *v*/v dilution at 4 °C overnight. After washed with TBST, membranes were incubated with Horseradish peroxidase (HRP)-conjugated sheep anti-mouse IgG (GE Healthcare Life Sciences, NA931-1ML) at room temperature for 1 h. After washed with TBST, the membranes were developed with Immobilon Western Chemiluminescent HRP Substrate (MERCK Millipore) and the signal was analyzed by Bio-Rad ChemiDocTM MP Imaging system.

### Animal immunization and lethal challenge experiments

All animal experiments were approved by the Committee on the Use of Live Animals in Teaching and Research at the Laboratory Animal Unit of the University of Hong Kong (CULATR 3569–15).

5- to 6- week-old male BALB/c mice were purchased from the Laboratory Animal Unit of the University of Hong Kong, and were kept under specific-pathogen-free conditions. The immunization procedure was similar to that described previously [26]. Briefly, BALB/c mice received three doses of S. Typhimurium vaccine by oral gavage on Day 1, Day 8 and Day 22. 4 h after mice were deprived of food and water, 100 μL 3% NaHCO3 was provided orally to neutralize the gastric acid. Then each mouse was vaccinated by 5 + E10 colony form unit (CFU) of freshly cultured and PBS-washed bacterial cells. The vaccination was conducted with a feeding needle with round bottom.

Ten days after the secondary booster, immunized mice were challenged through intravenously injecting 5E + 07 CFU of *S. aureus* USA 300 strain (community-associated MRSA [CA-MRSA]) or 5E + 07 CFU of Newman strains (methicillin-susceptible *S. aureus* [MSSA]). Mortality and clinical signs of mice were monitored for 14 days.

Animal experiments were repeated at least twice with similar results.

### Enzyme-linked immunosorbent assay (ELISA)

Seven days after the second booster, ~ 100 μL blood was collected from the tail vein using Microvette® CB 300 LH (Sarstedt), and stored at room temperature for 1 h, followed by centrifugation at 2000 g for 10 min at room temperature. Sera were collected for ELISA and could be stored at − 80 °C until use. Meanwhile, ~ 70 mg of feces were collected and suspended in 400 μL PBS. Then the mixture was vortexed and centrifuged at 10000 g for 15 min and the supernatants were collected for ELISA or stored at − 80 °C.

ELISA was performed as previously described [22]. Briefly, ELISA plates (Nuc, Roskilde, Denmark) were coated with recombinant SaEsxA (rSaEsxA) or rSaEsxB at the concentration of 1 μg/mL in coating buffer (carbonate-bicarbonate, pH = 9.6) overnight at 4 °C. Then ELISA plates were blocked with 5% *w*/*v* skim milk/TBST at 37 °C for 2 h. Mouse sera were serially diluted with 5% w/v skim milk/TBST at threefold and were added into the wells. While fecal extracts were serially diluted at twofold. After incubating for 1 h at 37 °C, the plates were washed five times with TBST. HRP-rabbit anti-mouse IgG2a secondary antibody (Invitrogen, 610,220), HRP-rabbit anti-mouse IgG1 secondary antibody (Thermo Scientific, PA1–86329) and HRP-goat anti-mouse IgA secondary antibody (Invitrogen, 62–6720) were used according to the manufacturer’s instructions. After incubating for 1 h at 37 °C, the plates were washed six times with TBST. For color development, plates were incubated with tetramethylbenzidine (TMB) substrate solution for 15 min at room temperature, followed by adding stop solution (2 M H_2_SO4). Absorbance at 450 nm was recorded by ELISA plate reader. The antibody titre was expressed as the inverse of the greatest dilution of sera which showing over twofold OD450 readout than that of the control sample at the same dilution. The assays were conducted in duplicate and the experiment were repeated twice.

### Enzyme-linked immunospot (ELISPOT) assay

Eight or nine days after the second booster, mice (3–4 mice/group) were euthanized with Pentobarbitone sodium (100–150 mg/kg, i.p.) and spleens were harvested, homogenised and forced through 70 μm cell strainer (BD) with the ends of sterile syringe plungers [45]. The splenocytes were obtained by centrifugation at 250 g. Erythrocytes were lysed with ACK buffer for 5 min at room temperature. After washing once with PBS, splenocytes were resuspended in RPMI 1640 medium with 10% FBS, and filtered with 70 μm cell strainer (BD) to get rid of cell aggregates.

Interferon-gamma (IFN-γ) and Interleukine-17A (IL-17A) ELISPOT assays were conducted with Mouse IFN-γ ELISPOT kit (R&D Systems) and Mouse IL-17 ELISpot Kit (R&D Systems) respectively, according to the manufacturer’s instructions. Briefly, splenocytes, stimulated by antigen rSaEsxA or rSaEsxB at the concentration of 2 μg/mL, were plated at the concentration of 5E + 05 cells/well in duplicate for 20 h at 37 °C. Then plates were washed and incubated with biotinylated anti-IFN-γ or anti-IL-17A antibody overnight at 4 °C. After washing the plates, streptavidin-Alkaline phosphatase was added and incubated for 2 h at room temperature. At last, the plates were incubated with substrate BCIP/NBT Chromogen for 0.5-1 h at room temperature for color development. The spots were counted using an immunospot reader system.

### Statistical analysis

Data were presented as the means ± SEM. For ELISPOT assays, the statistical significance was determined by Student’s *t* test. For survival rates in the lethal challenge experiments, statistical significance was determined by Log rank (Mantel-Cox) analysis. GraphPad Prism 6 was used to conduct these analyses. And a *P* value of < 0.05 was considered statistically significant.

## Results

### SPI-1 T3SS-dependent secretion of SaEsxA and SaEsxB

**Fig. 1:**
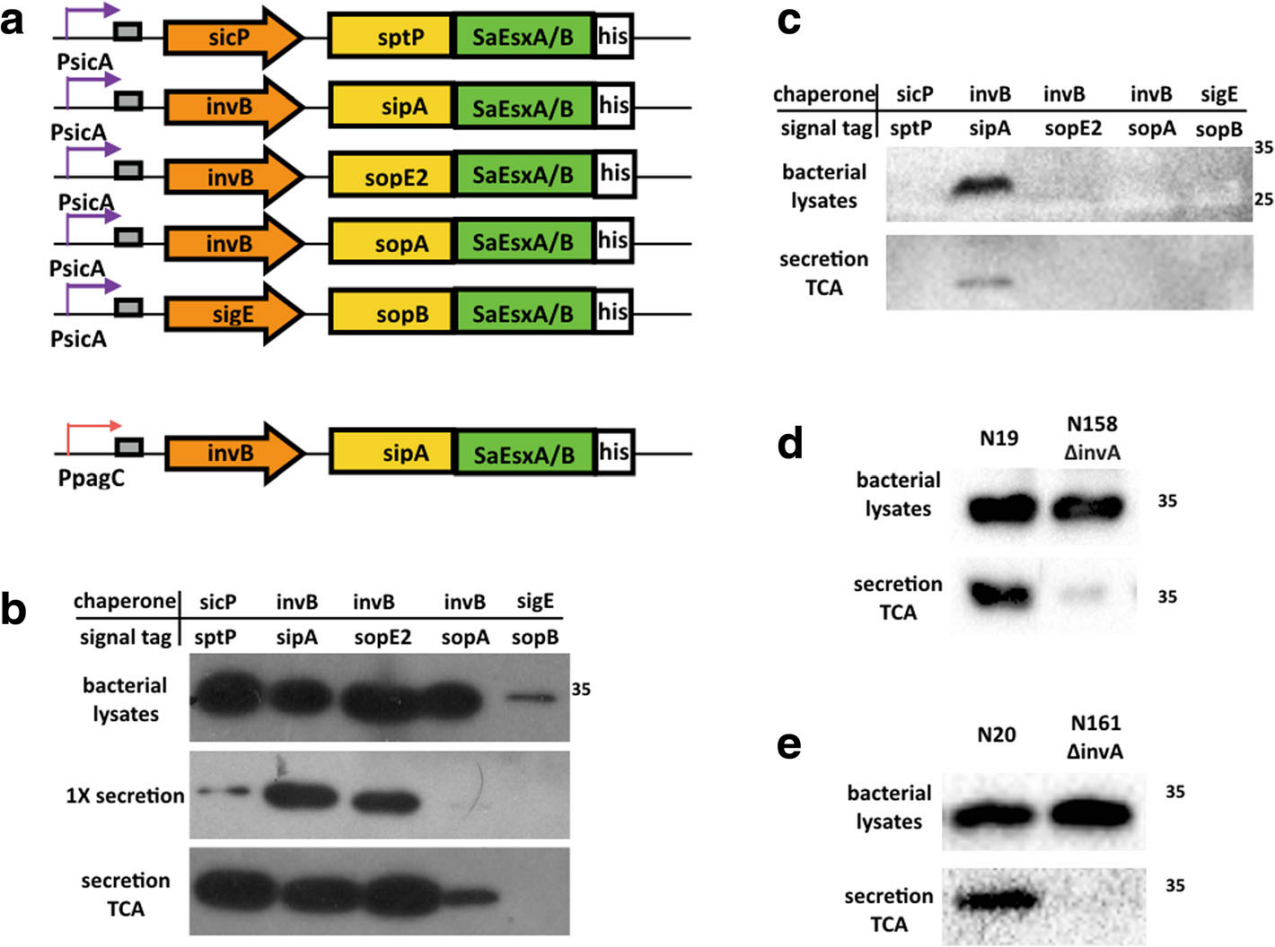
SPI-1 T3SS-dependent secretion of SaEsxA and SaEsxB fusing with different secretion tags. SaEsxA and SaEsxB were fused with N-terminal secretion tags, and were expressed with cognate chaperones under PsicA promoter. 6 × his tag at C-terminus was used for detection (**a**). Secretion assays were conducted for SaEsxA (**b**) and SaEsxB (**c**) to identify the optimal secretion tag/chaperone pairs. And the secretion of SipA-SaEsxA (**d**) and SipA-SaEsxB (**e**) from *S.* Typhimurium strain ML21 and isogenic ΔinvA mutant was compared. For bacterial cell lysates, an equivalent of 200 μL bacterial culture was used for SDS-PAGE. And for “1× secretion”, an equivalent of 13 μL culture was used and exposure time of western blot extended to 1 min. For “secretion TCA”, secreted proteins were further 20-fold concentrated by TCA precipitation, and an equivalent of 400 μL culture was used

Attenuated *S.* Typhimurium strains were engineered to express and secret antigens SaEsxA and SaEsxB via SPI-1 T3SS. As shown in Fig. 1a, SaEsxA and SaEsxB were fused with five different N-terminal secretion peptides of SPI-1 effectors under PsicA promoter, which is the endogenous promoter of SPI-1 effector sipA. Basal transcription level of PsicA is quite low, however upon induction with high osmolarity and low aeration, SPI-1 T3SS is established and activated [46], with a 200-fold increase of transcription efficiency [36]. His-tag at the C-terminal of antigens was used for detection purpose. Additionally, previous articles indicated the importance of specific chaperones of the secretion tags for secretion and translocation efficiency [29, 36], thus cognate chaperones was co-expressed with fusion antigens under PsicA.

To investigate the secretion efficiency of heterologous antigens, secretion assays were conducted, in which attenuated *S.* Typhimurium strain ML21 carrying a series of plasmids was cultured in low salt LB, and shifted to high salt LB media (0.3 M NaCl) to induce the secretion via SPI-1 T3SS. Expression and secretion were examined in vitro by western blot. Fig. 1b showed that all the five fusion proteins could be detected in bacterial cell lysates. Secretion efficiency of SipA-SaEsxA was comparatively high, which could be detected without TCA precipitation. After TCA precipitation, other SaEsxA fusion proteins were also detected in the LB media. For SaEsxB, among all five SaEsxB fusion proteins with different signal peptides, only SipA-SaEsxB was detected to be expressed and secreted from *S.* Typhimurium in inducing LB after TCA precipitation (Fig. 1c). Thus, the most optimal chaperon/tag pair for SaEsxA and SaEsxB was invB/sipA, which was utilized for further vaccine development. The expression and secretion level of SipA-SaEsxA is much higher than that of SipA-SaEsxB (data not shown). In order to activate potent immune responses in vivo, the SPI-1 T3SS-inducible promoter PsicA was replaced with in vivo-inducible promoter of phoP-activated gene C, designated as PpagC, which could lead to stable and increased expression of foreign antigens, and efficiently enhance immunogenicity in mice [47].

In order to demonstrate that the secretion of SaEsxA and SaEsxB is dependent of SPI-1 T3SS, rather than unspecific bacterial cell lysis, isogenic *S.* Typhimurium ΔinvA mutant ML88 was constructed for secretion assay. Gene invA encodes a critical structural component of SPI-1 T3SS, thus ΔinvA mutant is defective in SPI-1-mediated secretion [44]. As shown in Fig. 1d and Fig. 1e, secretion of SaEsxA and SaEsxB was eliminated in ΔinvA strain, although fusion proteins could be detected in the bacterial cell lysates. The ghost band in Fig. 1d might be the target protein released from lysed bacterial cells, rather than actively secreted via T3SS. Taken together, these data indicated that the protein can be secreted in a SPI-1 T3SS-dependent way.

### Cytosolic delivery of SaEsxA and SaEsxB into macrophages

**Fig. 2:**
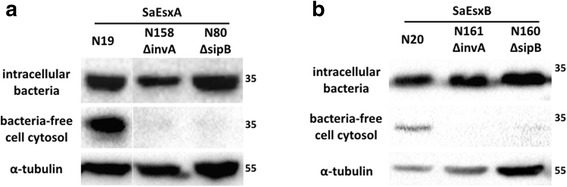
Translocation of SaEsxA and SaEsxB fusion proteins into macrophages. Raw264.7 macrophages were infected with ML21, isogenic ΔinvA mutant and ΔsipB mutant, carrying plasmids encoding SipA-SaEsxA (**a**) and SipA-SaEsxB (**b**) for 3 h. After lysis with 0.1% Triton X-100, infected macrophages were separated into Triton X 100-insoluble fraction containing intracellular bacteria and Triton X 100-soluble fraction containing bacteria-free cell cytosol. α-tubulin was used as an internal control for protein loading

To explore the potential of *S.* Typhimurium to translocate heterologous antigens into the cytosol of host cells, Raw264.7 macrophages were infected with *S.* Typhimurium strain ML21, ΔinvA mutant ML88 and ΔsipB mutant ML86, carrying plasmids encoding SipA-SaEsxA and SipA-SaEsxB. Gene sipB encodes one of the three translocators of SPI-1 T3SS. SipB is indispensable for the translocation of the effectors into the cytosol of host cells [48], which is as same as gene invA. After infection at multiplicity of infection (MOI) of 100 for 3 h, the infected macrophages were lysed with 0.1% Triton X-100. Presence of foreign antigens in both Triton X-100-insoluble fraction containing intracellular bacteria and Triton X-100-soluble fraction containing bacteria-free cytosolic proteins were examined by western blot. As shown in Fig. 2a, fusion protein SipA-SaEsxA was detected in the intracellular bacteria for *S.* Typhimurium strain N19, N158 (ΔinvA) and N80 (ΔsipB), which are ML21, ΔinvA mutant and ΔsipB mutant harboring plasmid encoding SipA-SaEsxA. Whereas, translocation of SipA-SaEsxA into the cytosol of macrophages was only detected from strain N19, but not from N158 (ΔinvA) and N80 (ΔsipB). Similarly, expression of SipA-SaEsxB can be detected in the intracellular bacteria for *S.* Typhimurium strain N20, N161 (ΔinvA) and N160 (ΔsipB), however translocation of SipA-SaEsxB was only detected in the bacteria-free cytosol of macrophages after infection with *S.* Typhimurium strain N20 (Fig. 2b). Thus, heterologous antigens SaEsxA and SaEsxB in fusion with N-terminal SipA (1-169aa) could be translocated into the cytosol of infected cells, which is dependent on SPI-1 T3SS. This data also indicates that N-terminal secretion and translocation domain of SipA could serve as a molecular carrier for cytosolic delivery of heterologous antigens, mediated by chaperone invB.

### Stimulation of antigen-specific humoral immune responses

**Fig. 3:**
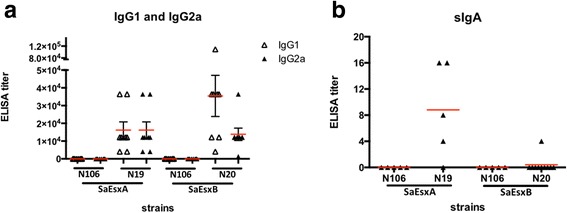
Evaluation of humoral and mucosal immune responses against rSaEsxA and rSaEsxB by ELISA. Mice were immunized with N106, N19 and N20 strains (*n* = 8/group). SaEsxA and SaEsxB were delivered via SPI-1 T3SS by N19 and N20, respectively. Seven days after the second booster, antigen-specific serum IgG1 and IgG2a (**a**), as well as fecal sIgA (**b**) were examined

To study the immunogenicity of *S.* Typhimurium-based vaccine in vivo, BALB/c mice were immunized with strain N19, N20 and the vector control strain N106, by oral gavage on Day 1, Day 8 and Day 22. Seven days after the 2nd booster, blood was collected and antigen-specific humoral immune responses in sera were examined by ELISA. As shown in Fig. 3a, mice immunized with N19 generated high level of rSaEsxA-specific IgG1 and IgG2a antibodies (average titers: 16,000), indicating that vaccination with N19 stimulated both Th1- and Th2-type responses. The rSaEsxB-specific IgG1 antibody titer of mice immunized with N20 was approximately 37,000, while rSaEsxB-specific IgG2a antibody titer was approximately 13,000. This result suggested that SaEsxB delivered by *S.* Typhimurium vaccine stimulated Th2-biased immune responses.

Considering the importance of secretory IgA (sIgA) in immune exclusion and homeostasis maintenance of *S. aureus* in the intestine tract [49], sIgA antibodies against rSaEsxA and rSaEsxB in fecal extract of vaccinated mice were also determined by ELISA. The results indicated the presence of anti-rSaEsxA sIgA in the fecal extract of 4/5 mice vaccinated with N19. While for the mice vaccinated with N20, anti-rSaEsxB sIgA was detected in only 1/10 mice (Fig. 3b). No antigen-specific antibodies were detected from the mice immunized with the control strain N106.

Raw data are available in Additional file 1: Table S1.

### Stimulation of antigen-specific IFN-γ^+^ and IL-17A^+^ T cell immune responses

**Fig. 4:**
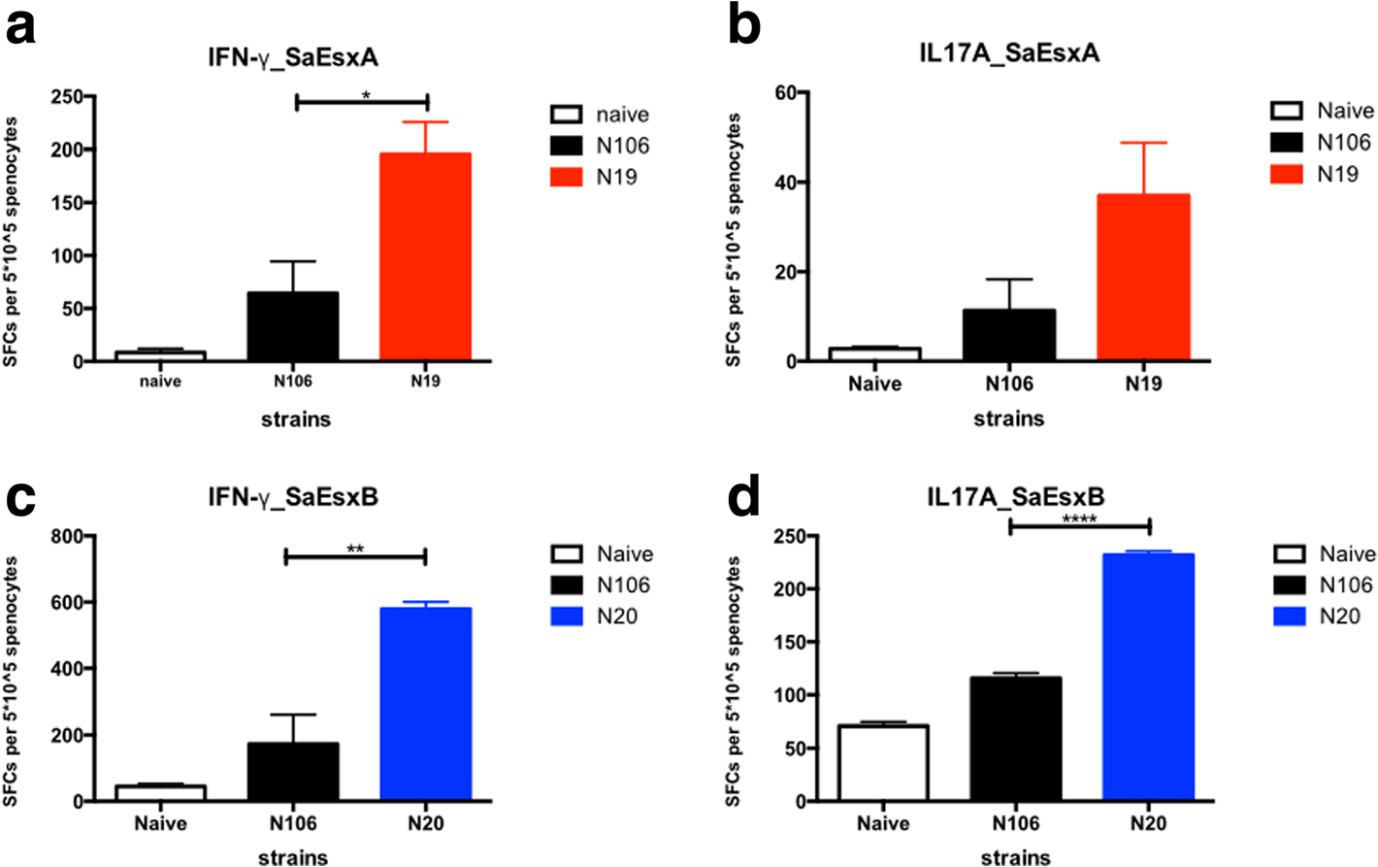
Evaluation of cellular immune response against rSaEsxA and rSaEsxB by ELISPOT. Mice were immunized with PBS, N106, N19, and N20 strains (*n* = 3–4/group). Eight or nine days after the second booster, splenocytes were prepared and incubated with specific antigens for 20 h. IFN-γ-producing cells with stimulator rSaEsxA (**a**) and rSaEsxB (**c**) were detected by IFN-γ ELISPOT assay. And for IL-17A-producing cells stimulated with rSaEsxA (**b**) and rSaEsxB (**d**), IL-17A ELISPOT assays were conducted. SFC, Spot-Forming Cells. Results of one of two experiments are shown. For statistical analysis, student’s t test was used. Data were represented as means ± SEM

Previous research indicated that Th1 and Th17 cell-mediated immunity contributes to the protection against *S. aureus* infection [50, 51], therefore we investigated whether SaEsxA and SaEsxB delivered by attenuated *S.* Typhimurium could elicit specific cellular immune responses. After the 2nd booster, IFN-γ and IL-17A ELISPOT assays were conducted with splenocytes collected from vaccinated mice. The result in Fig. 4a and 4b showed that after stimulated with rSaEsxA, splenocytes from mice vaccinated with N19 had more IFN-γ- and IL-17A-secreting cells than that of mice treated with control strain N106 and PBS. Similarly, mice immunized with N20 showed strong rSaEsxB-specific IFN-γ^+^ and IL-17A^+^ T cell responses (Fig. 4c and 4d). Taken together, these data suggest that vaccination by N19 and N20 strains could elicit antigen-specific Th1- and Th17-biased cellular immune responses.

Raw data are available in Additional file 2: Table S2.

### Protection against lethal challenge

**Fig. 5:**
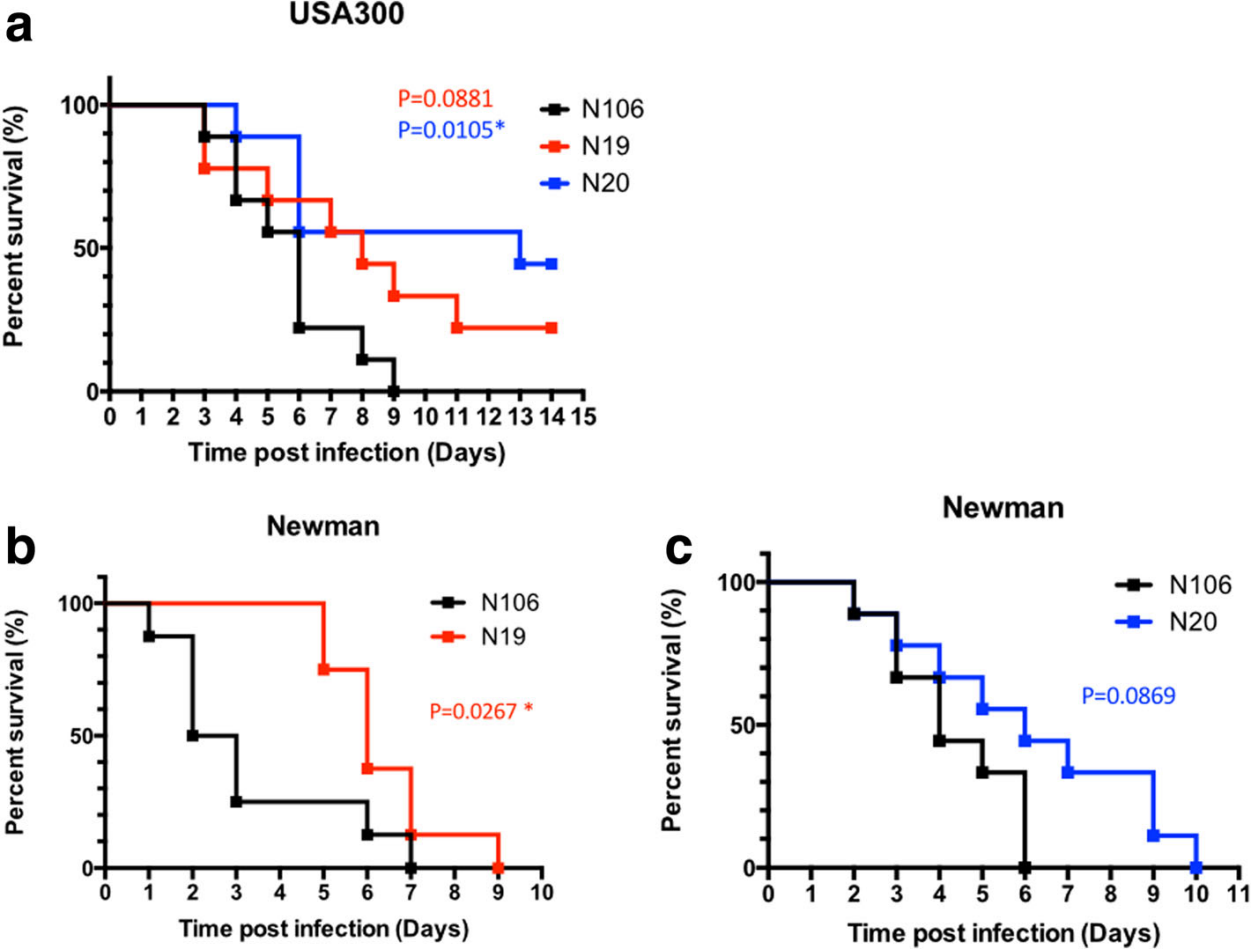
Survival curves of vaccinated BALB/c mice after lethal challenge with two clinical *S. aureus* strains. Mice immunized with N106, N19 and N20 were challenged with *S. aureus* USA 300 (**a**) or Newman strains (**b**, **c**)(5E + 07 CFU) by intravenous injection (*n* = 8–9/group). Mice were monitored for 14 days. Log rank (Mantel-Cox) tests were conducted to compare the protection induced by different vaccination. Results from one of two representative experiments are shown

To evaluate the protection efficacy conferred by different vaccinations, ten days after the second booster, vaccinated mice were challenged with two clinical *S. aureus* strains at 5E + 07 CFU by intravenous injection through the tail vein. Mice were monitored for 14 days. The results in Fig. 5a revealed that oral administration of N20 significantly reduced the death of mice after lethal challenge with *S. aureus* USA 300 (44.4% survival; *P* = 0.0105, log rank Mantel-Cox test), in comparison the mice in N106 control group. Whereas, vaccination with N19 improved the survival rate to 22.2% (*P* = 0.0881, log rank Mantel-Cox test). In addition, when challenged with *S. aureus* Newman strain, even though all mice died within 10 days, vaccination with N19 and N20 could stimulate protective effect and postpone death, as shown in Fig. 5b and 5c. (for N19, *P* = 0.0267; for N20 *P* = 0.0869, log rank Mantel-Cox test).

Raw data are available in Additional file 3: Table S3.

## Discussion

In this study, live attenuated *S.* Typhimurium vaccine against *S. aureus* was developed, in which staphylococcal antigens SaEsxA and SaEsxB were delivered into the cytosol of host cells via SPI-1 T3SS. Oral administration of these vaccines elicited multi-faceted immune responses, including both humoral and cell-mediated immune responses, which confer protection against two clinical *S. aureus* strains.

Specific antibodies including SaEsxA- and SaEsxB-specific IgG in serum, as well as SaEsxA-specific sIgA in fecal extract were detected in vaccinated mice. During earlier stage of *S. aureus* infection, initial elimination of extracellular *S. aureus* was largely dependent on opsonophagocytosis facilitated by specific antibodies and complement components in serum [52]. Those SaEsxA- and SaEsxB-specific IgG antibodies might be crucial for binding and neutralizing *S. aureus* while it is in the extracellular stage. However, previous study on protein subunit vaccines demonstrated that antibodies generated against SaEsxA and SaEsxB were not effective against *S. aureus* infection, suggesting that cellular immune responses may be important to achieve good efficacy [22].

Antigen-specific stimulation of IFN-γ secretion was detected in vaccinated mice, indicating the activation of Th1-type immune response. This data is consistent with previous reports that oral administration of *S.* Typhimurium vaccine elicited Th1-biased immune responses [1, 27, 53, 54], which effectively fight against intracellular pathogens [55, 56]. As accumulated evidence suggested, *S. aureus* is not exclusively extracellular bacterium, but is also capable of invading host cells. Upon invasion, the intracellular *S. aureus* can persist and replicate in the phagosomes, while some can escape from the phagosomes and enter the cytoplasm [57, 58]. The intracellular fate of *S. aureus* in professional phagocytes was postulated to form a dissemination route of infection [59]. Cytotoxic T cells, natural killer cells and cytokine IFN-γ are required to eliminate the intracellular bacteria [60, 61]. Therefore, the stimulated IFN-γ secretion could play important role in the clearance of intracellular *S. aureus*.

Th17 responses were also induced by *S.* Typhimurium delivering SaEsxA and SaEsxB, as suggested by the detection of IL-17A^+^ splenocytes. Many studies indicated the importance of Th17 responses in the protection against a wide range of bacterial and fungal pathogens [62]. Th17 cells could mediate serotype-independent protection, through several mechanisms, such as recruiting neutrophils and macrophages to mucosal sites, activating B cell antibody responses [63, 64]. Besides, long-life Th17 effector memory cells were detected in mucosal tissues, indicating their potential role against pathogens [65].

Previous researchers have shown that numerous viral and bacterial antigens were delivered into the cytosol of host cells via SPI-1 T3SS, presented to MHC class I-restricted antigen processing pathway, and subsequently activated CD8+ T cell-mediated immune responses [27, 29–31]. Our data showed the secretion and translocation of SipA-SaEsxA and SipA-SaEsxB fusion proteins into cytosol of Raw264.7 macrophages in vitro and stimulation of IFN-γ-dependent cellular immunity in vivo. Therefore, we hypothesized that the staphylococcal antigens delivered by *S.* Typhimurium carrying plasmids pCASP-invB-sipA-SaEsxA/B were presented by MHC class I molecules to CD8+ T cells, which could fight against *S. aureus* at intracellular stage.

Several limitations in the current study should be mentioned. Firstly, even though the two *S.* Typhimurium vaccines targeting either SaEsxA or SaEsxB could elicit significant antigen-specific immunity, neither of them conferred effective protection against the challenge with both *S. aureus* USA 300 and Newman strains. As suggested by previous articles that, in comparison with single-antigen vaccine, multivalent vaccines could evoke broad immune responses, and confer protection against different serotypes of *S. aureus*. Thus, to improve *S.* Typhimurium vaccine, a multivalent vaccine should be designed to facilitate the simultaneous delivery of SaEsxA, SaEsxB and other antigens. Secondly, antigen expression level in the recombinant attenuated *Salmonella* is one of the most fundamental determinants of vaccine efficacy. It’s observed that the expression level of SaEsxB in *S*. Typhimurium is comparatively low, thus codon optimization of SaEsxB should be conducted to ensure optimal amount of SaEsxB expressed and delivered into host cells to achieve more potent immunogenicity.

## Conclusions

In summary, this study suggested that SPI-1 T3SS could be exploited for cytosolic delivery of staphylococcal antigens SaEsxA and SaEsxB, to effectively activate antigen-specific humoral and cellular immune responses, and provide protection against *S. aureus* infection.

## Additional files

Additional file 1: **Table S1.** Evaluation of humoral and mucosal immune responses against rSaEsxA and rSaEsxB by ELISA. Readout at OD450 was shown. (XLSX 49 kb)

Additional file 2: **Table S2.** Evaluation of cellular immune response against rSaEsxA and rSaEsxB by ELISPOT. The spot-forming cells were counted using an immunospot reader system. The spot numbers were shown. (XLSX 41 kb)

Additional file 3: **Table S3.** Survival curves of vaccinated mice after lethal challenge with two clinical *S. aureus* strains. Mouse numbers were shown after challenge with *S. aureus* strains. (XLSX 35 kb)

## Abbreviations

APCs: Antigen presenting cells
BHI: Brain Heart Infusion Broth
CA-MRSA: Community-associated MRSA
CFU: Colony forming unit
ELISPOT: Enzyme-linked immunospot
ESAT-6: Early Secreted Antigenic Target-6
FBS: Fetal bovine serum
HRP: Horseradish peroxidase
IFN-γ: Interferon-gamma
IL-17A: Interleukine-17A
LB: Luria-Bertani
*M. tuberculosis*: *Mycobacteria tuberculosis*
MOI: Multiplicity of infection
MRSA: Methicillin-resistant *S. aureus*
MSSA: Methicillin-susceptible *S. aureus*
PEEC: Pathogen-elicited epithelial chemoattractant
PKC: Protein kinase C
PpagC: PagC promoter
*S. aureus*: *Staphylococcus aureus*
*S.* Typhimurium: *Salmonella enterica* subsp. *enterica* serotype Typhimurium
SFC: Spot-forming cells
sIgA: Secretory IgA
SPI-1 T3SS: *Salmonella* Pathogenesis Island-1 Type 3 Secretion System
TCA: Trichloroacetic acid
TMB: Tetramethylbenzidine
